# White Fillings

**Published:** 1884-03

**Authors:** 


### White Fillings.

A dentist wishes to know the formula for the white cement used for filling decayed teeth, which contains neither mercury nor silver in its composition.

There are several white fillings in use by dental surgeons which contain neither murcury nor silver. They are made by mixing oxide of zinc with impalpable glass powder in small proportion, and just before using, when the cavity of the tooth is prepared, a small quantity of deliquesced chloride of zinc is placed on a glass slab, and enough powder added to make a thick paste, mixed rapidly. It "sets" very quickly, and forms a good temporary stopping. It is slightly irritating to the "nerve" of a tooth, and should not be inserted directly in a cavity in which caries has far advanced without placing a little solution of gutta-percha in chloroform over the region of the pulp. But a less irritating filling, according to the London *Lancet*, is made by mixing the same powder of oxide of zinc with pyrophosphoric acid; this is a more permanent white stopping.

